# Transcriptomic and intervention evidence reveals domestic dogs as a promising model for anti‐inflammatory investigation

**DOI:** 10.1111/acel.14127

**Published:** 2024-03-01

**Authors:** Min Zeng, Tong Zhou, Zhiyu Li, Guimei Li, Shurun Zhang, Lu Wang, Qing‐Guo Huang, Ju‐Dong Li, P. Nadeeshika Samarawickrama, Yonghan He, Guo‐Dong Wang

**Affiliations:** ^1^ Key Laboratory of Genetic Evolution & Animal Models Kunming Institute of Zoology, Chinese Academy of Sciences Kunming China; ^2^ Kunming College of Life Science University of Chinese Academy of Sciences Kunming China; ^3^ State Key Laboratory for Conservation and Utilization of Bio‐Resources in Yunnan Yunnan University Kunming China; ^4^ Kunming Police Dog Base of the Chinese Ministry of Public Security Kunming China; ^5^ Key Laboratory of Healthy Aging Research of Yunnan Province Chinese Academy of Sciences Kunming China; ^6^ Yunnan Key Laboratory of Molecular Biology of Domestic Animals Chinese Academy of Sciences Kunming China

**Keywords:** age‐associated genes, aging, anti‐inflammatory, canine mesenchymal stem cells, dog models

## Abstract

Domestic dogs have great potential to expand our understanding of the determinants of aging. To understand the aging pattern of domestic dogs and evaluate whether they can be used as an aging model, we performed RNA sequencing on white blood cells from domestic dogs aged 1–9 years and treated aged dogs with classical antiaging approaches. We obtained 30 RNA sequencing libraries and identified 61 age‐associated genes with dynamic changes, the majority of which were related to metabolism and immune function, which may be predominant biomarkers for aging in dogs. We next treated aged dogs with canine mesenchymal stem cells (cMSCs), nicotinamide mononucleotide, and rapamycin to determine whether and how they responded to the antiaging interventions. The results showed that these treatments can significantly reduce the level of inflammatory factors (IL‐6 and TNF‐α). MSCs effectively improved the heart functions of aged dogs. Three key potential age‐related genes (*PYCR1, CCRL2*, and *TOX*) were reversed by MSC treatment, two of which (*CCRL2* and *TOX*) are implicated in immunity. Overall, we profiled the transcriptomic pattern of domestic dogs and revealed that they may be a good model of aging, especially in anti‐inflammatory investigations.

## Graphical abstract

We profiled age‐related transcriptome of domestic dogs and found that inflammation‐associated genes are significantly differentially expressed. We further treated aged dogs with canine mesenchymal stem cells (MSCs), nicotinamide mononucleotide, and rapamycin, all of which can significantly reduce the proinflammatory factors in plasma (e.g., IL‐6 and TNF‐α). Of note is that MSCs effectively improved the heart function and reversed the expression of some age‐ and inflammation‐related genes in aged dogs, suggesting domestic dogs as a promising model for anti‐inflammatory investigation.

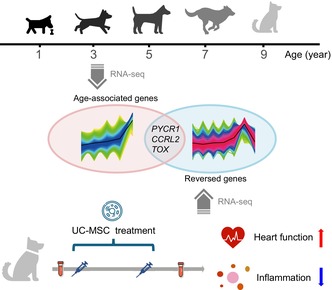

**Abbreviations**
A‐AGs: age‐associated genes
cMSCs: canine mesenchymal stem cells
DAP: Dog Aging Project
DEGs: differentially expressed genes
GO: Gene Ontology
HRR: heart rate reserve
HRT: heart rate
IGF‐1: insulin‐like growth factor‐1
IL‐6: interleukin‐6
KEGG: Kyoto Encyclopedia of Gene and Genomes
NMN: nicotinamide mononucleotide
Rapa: rapamycin
TNF‐α: tumor necrosis factor α

## 1 INTRODUCTION

Biological aging is a complex process with 12 hallmarks that are interconnected (Lopez‐Otin et al., 2023). Animal models provide the opportunity to decelerate, stop, or reverse aging through therapeutic interventions on these hallmarks. Classical models of aging, such as mouse, rat, fruit fly, and roundworm models, are well established and short‐lived, but their specific characteristics may result in inappropriate generalizations, which may cause interpretation biases (Holtze et al., 2021). Rhesus macaques are considered the most appropriate model for human beings, but the costs, ethical concerns, legal considerations, species conservation considerations, lifespan, and supply limit the contribution of nonhuman primates. Therefore, additional models mimicking human aging are greatly needed.

Dogs are an attractive model because they spontaneously develop many aging‐related phenotypes (Ruple et al., 2022), have undergone convergent evolution with humans (Cao et al., 2021; Liu et al., 2021; Wang, Zhai, et al., 2013; Zhou et al., 2022), and have similar survivorship curves to humans (Hoffman et al., 2018). Moreover, dog models may overcome many limitations of laboratory models because they share similar environments and lifestyles with humans (Ruple et al., 2022). Furthermore, abundant resources enable research on dog models of aging. The Dog Aging Project (DAP) provides data about the biological and environmental determinants of aging and medical records (Kaeberlein et al., 2016), and the Golden Retriever Lifetime Study is a longitudinal study of more than 3000 Golden Retrievers over their lifetimes with a focus on cancer. Some experiments that failed in mouse models may be performed in dog models, such as those on cancer and blood glucose metabolism (Hoffman et al., 2018; Palliyaguru et al., 2021).

Aging is a complex process that includes frailty, multimorbidity, and syndromes of aging. Frailty may represent a transition phase between successful aging and disability, and it appears to be more predictive of death (Cesari et al., 2016). Many interventions have been shown to increase lifespan or healthspan in laboratory mouse models that may be suitable for translational geroscience applications in dogs or people (Kaeberlein et al., 2016). The DAP offers the opportunity to test interventions to delay the impact of aging, and they carried out a study on rapamycin (Rapa) (Barnett et al., 2023; Kaeberlein et al., 2016; Urfer et al., 2017). Rapa is an mTOR inhibitor that can extend the lifespan in mouse models with immunosuppressive properties (Neff et al., 2013) and has great potential for suppressing multimorbidity in old age. Nicotinamide mononucleotide (NMN) (a precursor of NAD+) is downregulated with age and commonly used in antiaging research, which makes it an attractive target for therapeutic intervention in degenerative disorders (Mills et al., 2016). In addition, stem cell therapy may be effective in frailty, and it has emerged as a promising breakthrough not only for treating complex diseases but also as a potential intervention to combat aging (Zhu, Ge, et al., 2021). To test whether dogs respond to these anti‐aging approaches, we treated aged dogs with canine mesenchymal stem cells (cMSCs), NMN, and Rapa, which can help us gain more insights into the determinants of aging.

To further promote the development and practical application of dog models, we collected blood samples from dogs aged 1–9 to explore blood marker genes during the dog aging process. Then, we cultured cMSCs from the umbilical cord as a cell therapy intervention in aged dogs. In addition, we selected Rapa and NMN for comparison. We identified 61 aging‐related genes, 10 of which were mainly related to immunity. The therapeutic efficacy of our treatments was also focused on immunity. Our study provides evidence that domestic dogs may act as a good model to study the influence of inflammation on aging.

## 2 RESULTS

### 2.1 Transcriptomic baseline and age‐associated genes in domestic dogs

**FIGURE 1:**
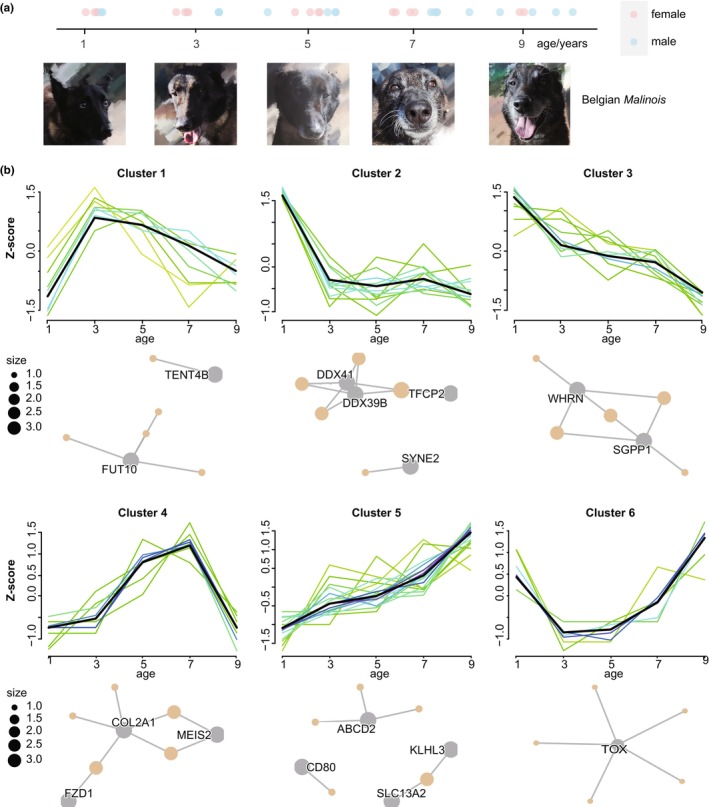
Clusters of 61 age‐associated genes. (a) Thirty Belgian *Malinois* for transcriptomic baseline. (b) Sixty‐one age‐associated genes were identified, and these genes were grouped into six clusters with different patterns. The Z score (y‐axis) was used to show relative changes in gene expression at different ages (x‐axis). The cnetplot shows the plot linkages of key genes and enriched concepts.

We collected 30 dogs from the Kunming Police Dog Base of the Ministry of Public Security, and they were kept under the same conditions. The participants were aged 1, 3, 5, 7, and 9 years (rounded by year) (Figure 1a). Detailed information is shown in Table 1. Blood samples were obtained and submitted for whole blood RNA‐Seq for dynamic expression analysis. To analyze the dynamic changes in gene expression with aging, we grouped all genes by their expression profiles across a series of ages using the Mfuzz R package (Kumar & Futschik, 2007); canine, human, and mouse one‐to‐one orthologs were used as the candidate genes (OMA Orthology Database, hemoglobin genes removed). These genes were finally grouped into 8 clusters (Figure S1a). Genes showed striking expression changes at Ages 3 and 7, which may be associated with the physical maturity and presenium/senectitude of large‐sized dogs, as their bodies mature at approximately 22–24 months. Gene expression in Clusters 2, 3, 4, 6, 7, and 8 changed abruptly from Age 3 in adolescence, while Clusters 2, 3, 4, 5, and 6 changed abruptly at Age 7 in presenium.

**TABLE 1:** Information of dogs.

Sample_ID	Name	Chip_number	Sex	Age	Chronological_age	Birthdate	Breed	Note	Batch
BM1001	Bate	119920	M	10	9.75	20120423	BM	Treated group	1
BM1002	Piliqi	2873953	M	9	9.45	20120808	BM	Treated group	1
BM1005	Aikesi	2241688	M	9	8.80	20130402	BM		1
BM0001	Fuerkuai	5009707	F	9	9.10	20121209	BM		1
BM0002	Xijiashi	5009609	F	9	9.03	20130104	BM		1
BM1003	Lunchengfa	8023504	M	8	8.45	20130808	BM	Not target age	1
BM1004	Lunmengsheng	3219584	M	8	7.90	20140226	BM	Not target age	1
BM1006	Zhengchengxing	3219988	M	7	7.32	20140927	BM		1
BM1007	Yuannixing	8021090	M	7	7.27	20141012	BM		1
BM1008	Ruopishun	3208272	M	7	7.21	20141104	BM		1
BM1009	Qingdishi	8019173	M	7	7.21	20141106	BM		1
BM0004	Shanizun	8019166	F	7	7.19	20141107	BM		1
BM0005	Pasilute	209992	F	7	6.82	20150321	BM		1
BM0006	Renlunshan	8022910	F	7	6.56	20150622	BM		1
BM0003	Chuannazuo	8019096	F	7	6.51	20150712	BM		1
BM1011	Saipite	8055179	M	5	5.00	20170119	BM		1
BM1012	Dongzhenbai	8056974	M	5	4.99	20170121	BM		1
BM1013	Xilunri	8057001	M	5	4.67	20170521	BM		1
BM0008	Yuanbayue	8052955	F	5	5.49	20160718	BM		1
BM0009	Donglunwo	8051550	F	5	5.49	20160719	BM		1
BM0007	Nachengshi	8058315	F	5	5.18	20161109	BM	Doubtful sample	1
BM0010	Shidanpi	8051041	F	5	4.97	20170124	BM		1
BM1010	Airuisi	1522211	M	4	3.88	20180305	BM	Not target age	1
BM1014	Zhimahu	8053187	M	3	3.41	20180821	BM		1
BM1015	Junixia	8056268	M	3	3.41	20180823	BM		1
BM1016	Zuobaxu	8052316	M	3	3.31	20180927	BM		1
BM1017	Qingjiantian	8054244	M	3	3.27	20181014	BM		1
BM0012	Luyuanzhai	8057895	F	3	3.32	20180917	BM	Doubtful sample	1
BM0013	Pula	144987	F	3	3.21	20181028	BM		1
BM0014	Shanjiandeng	8057535	F	3	3.20	20181101	BM	Doubtful sample	1
BM0011	Juelunjiao	8058288	F	3	2.85	20190309	BM		1
BM1018	Yousuode	8081235	M	1	1.33	20200921	BM		1
BM1019	Huangaide	8096727	M	1	1.03	20210106	BM		1
BM0015	Zhusuokuai	8098165	F	1	1.29	20200928	BM		1
BM0016	Goudishang	8098277	F	1	1.22	20201024	BM		1
BM0017	Yangchuomo	8090821	F	1	1.21	20201027	BM		1
BM0018	Kezhensi	8084439	F	1	1.17	20201111	BM		1
1	Mafengchao	8021995	M	8	8.23	20121114	KD	MSC	2
2	Jingeke	5009763	M	8	8.38	20120923	KD	NMN	2
3	Yinglianqiao	8022121	M	8	8.35	20120104	KD	MSC	2
4	Seseling	8022092	M	8	8.36	20120930	KD	MSC	2
5	Lueluolian	8025910	M	9	8.56	20120716	KD	NMN	2
6	Dijiabo	8020319	M	9	8.65	20120614	BM	MSC	2
7	Nansewang	8021909	M	9	8.83	20120411	KD	Rapa	2
8	Zhennafu	8013853	M	10	10.37	20100925	BM	MSC	2
9	Piliqi	2873953	M	9	8.50	20120808	BM	Rapa	2
10	Jiuqinglue	8025844	M	9	8.58	20120712	KD	Fierce dog	2
11	Fengsexing	8025827	M	9	8.60	20120703	KD	MSC	2
12	Bate	119920	M	9	8.79	20120423	BM	NMN	2
13	Suokuaixiang	8025869	M	9	8.59	20120708	BM	Rapa	2
14	Tekuaimu	5006815	F	10	10.01	20110205	BM	NMN	2
15	Duokuaiwa	5008020	F	10	9.62	20110627	BM	Rapa	2
16	Kaanma	8020286	F	9	8.65	20120613	BM	NMN	2
17	Jingebo	5009765	F	8	8.38	20120923	KD	Rapa	2
18	Nanbaoxiong	8016985	F	11	10.58	20100712	KD	Rapa	2

*Note*: None of them were sterilized, pregnant and overweight/underweight.

Abbreviations: BM, Belgian *Malinois*; KD, Kunming dog; MSC, mesenchymal stem cells; NMN, nicotinamide mononucleotide; Rapa, rapamycin.

We next performed Gene Ontology (GO) analysis for the genes in each cluster separately (Table S1). Notably, gene expression in Cluster 5 was dramatically changed only at Age 7, and it was mainly enriched in adhesion and adaptive immune‐related GO terms, such as biological adhesion, cell adhesion, T‐cell differentiation, T‐cell activation, and regulation of immune response signaling pathways. This suggests that cell adhesion and inflammation‐related changes occur during presenium. Genes in Cluster 6 displayed a gradual decrease with age and were enriched in RNA, mRNA, tRNA, and ncRNA processing and metabolic processes.

### 2.2 Benchmark of age‐associated genes

To identify the key genes in aging, maSigPro (Nueda, 2021) was used to analyze dynamic gene profiles across the overall ages compared with ImpulseDE2 (Figure S1c). maSigPro is an R package that can identify the genes with dynamic expression profiles across the overall ages (Cardoso‐Moreira et al., 2019). We found 61 significant age‐associated genes (A‐AGs), and according to the expression profiles of the genes at different ages, these genes were grouped into six clusters using Mfuzz v2.52.0 software (Figure 1b, Table 2). None of the 61 genes overlapped with the sex‐related differentially expressed genes (DEGs) identified by DESeq2, indicating that these A‐AGs were not related to sex (Figure S1d, Table S2).

**TABLE 2:** 61 age‐associated genes.

Ensembl ID	Cluster	Symbol	Description
ENSCAFG00000020061	1	CERCAM	Cerebral Endothelial Cell Adhesion Molecule
ENSCAFG00000017350	1	ELAVL3	ELAV Like RNA Binding Protein 3
ENSCAFG00000016468	1	SEPSECS	Sep (O‐Phosphoserine) TRNA:Sec (Selenocysteine) TRNA Synthase
ENSCAFG00000013782	1	CCRL2	C‐C Motif Chemokine Receptor Like 2
ENSCAFG00000009938	1	TENT4B	Terminal Nucleotidyltransferase 4B
ENSCAFG00000006336	1	FUT10	Fucosyltransferase 10
ENSCAFG00000005906	1	PYCR1	Pyrroline‐5‐Carboxylate Reductase 1
ENSCAFG00000005585	1	MRPL48	Mitochondrial Ribosomal Protein L48
ENSCAFG00000030184	2	NANP	N‐Acetylneuraminic Acid Phosphatase
ENSCAFG00000016558	2	MTHFR	Methylenetetrahydrofolate Reductase
ENSCAFG00000016304	2	DDX41	DEAD‐Box Helicase 41
ENSCAFG00000015819	2	SYNE2	Spectrin Repeat Containing Nuclear Envelope Protein 2
ENSCAFG00000013699	2	LRWD1	Leucine Rich Repeats And WD Repeat Domain Containing 1
ENSCAFG00000013011	2	PDE8A	Phosphodiesterase 8A
ENSCAFG00000012301	2	TNS3	Tensin 3
ENSCAFG00000007831	2	TFCP2	Transcription Factor CP2
ENSCAFG00000001362	2	PLXNA4	Plexin A4
ENSCAFG00000000765	2	AGPAT4	1‐Acylglycerol‐3‐Phosphate O‐Acyltransferase 4
ENSCAFG00000000761	2	ATF6B	Activating Transcription Factor 6 Beta
ENSCAFG00000000509	2	DDX39B	DExD‐Box Helicase 39B
ENSCAFG00000032183	3	SGPP1	Sphingosine‐1‐Phosphate Phosphatase 1
ENSCAFG00000017070	3	TRMT1	TRNA Methyltransferase 1
ENSCAFG00000007601	3	ZNF536	Zinc Finger Protein 536
ENSCAFG00000004729	3	NUP85	Nucleoporin 85
ENSCAFG00000003758	3	PCDH18	Protocadherin 18
ENSCAFG00000003344	3	WHRN	Whirlin
ENSCAFG00000002050	3	CHIC2	Cysteine Rich Hydrophobic Domain
ENSCAFG00000001426	3	COPG2	COPI Coat Complex Subunit Gamma 2
ENSCAFG00000000454	3	VARS2	Valyl‐TRNA Synthetase 2, Mitochondrial
ENSCAFG00000032367	4	MPV17	Mitochondrial Inner Membrane Protein MPV17
ENSCAFG00000018123	4	ARSI	Arylsulfatase Family Member I
ENSCAFG00000009448	4	SLC10A6	Solute Carrier Family 10 Member 6
ENSCAFG00000009180	4	GPR15	G Protein‐Coupled Receptor 15
ENSCAFG00000009059	4	COL2A1	Collagen Type II Alpha 1 Chain
ENSCAFG00000008498	4	MEIS2	Meis Homeobox 2
ENSCAFG00000008350	4	PKIA	CAMP‐Dependent Protein Kinase Inhibitor Alpha
ENSCAFG00000001906	4	FZD1	Frizzled Class Receptor 1
ENSCAFG00000028762	5	KLRG1	Killer Cell Lectin Like Receptor G1
ENSCAFG00000018719	5	ALDOC	Aldolase, Fructose‐Bisphosphate C
ENSCAFG00000018692	5	SLC13A2	Solute Carrier Family 13 Member 2
ENSCAFG00000018379	5	GZMK	Granzyme K
ENSCAFG00000013122	5	MAGI3	Membrane Associated Guanylate Kinase, WW And PDZ Domain Containing 3
ENSCAFG00000012478	5	PLEKHA5	Pleckstrin Homology Domain Containing A5
ENSCAFG00000011178	5	CD160	MUSSI
ENSCAFG00000010997	5	CD80	CD80 Molecule
ENSCAFG00000010458	5	SHANK2	SH3 And Multiple Ankyrin Repeat Domains 2
ENSCAFG00000010022	5	ABCD2	ATP Binding Cassette Subfamily D Member 2
ENSCAFG00000009427	5	PTPN13	Protein Tyrosine Phosphatase Non‐Receptor Type 13
ENSCAFG00000005731	5	NRG2	Neuregulin 2
ENSCAFG00000004408	5	CAB39L	Calcium Binding Protein 39 Like
ENSCAFG00000003959	5	RAB19	RAB19, Member RAS Oncogene Family
ENSCAFG00000002741	5	MYO6	Myosin VI
ENSCAFG00000002023	5	RCAN2	Regulator Of Calcineurin 2
ENSCAFG00000001646	5	PRUNE2	Prune Homolog 2 With BCH Domain
ENSCAFG00000001126	5	KLHL3	Kelch Like Family Member 3
ENSCAFG00000023806	6	KRT25	Keratin 25
ENSCAFG00000007158	6	SLC16A12	Solute Carrier Family 16 Member 12
ENSCAFG00000007137	6	TOX	Thymocyte Selection Associated High Mobility Group Box
ENSCAFG00000006374	6	C24H20orf194	Dynein Axonemal Assembly Factor 9
ENSCAFG00000003242	6	BSPRY	B‐Box And SPRY Domain Containing
ENSCAFG00000000810	6	TRPS1	Transcriptional Repressor GATA Binding 1

Gene expression in Cluster 1 (Figure 1b) decreased after age 3 and has been shown to be involved in developmental programs; for instance, *FUT10* is predicted to contribute to cortical cell migration and neuronal stem cell division. Genes in Cluster 2 remained stable after 3 years of age and influenced RNA splicing and transesterification reactions. Cluster 3 gradually decreased with age and was involved in epidermal cell differentiation, while Cluster 5 increased with age and affected peroxisomal membrane transport, lipid catabolic processes, sodium ion transport, and T‐cell costimulation. Genes in Cluster 4 play roles in ossification; cell aggregation; and notochord, head, and sensory organ development; expression of these genes increases with age but declines after age 7. Gene expression in Cluster 6 gradually increased after 3 years of age; *TOX* may be the most influential gene in this cluster, regulating T‐cell lineage commitment, T‐cell and NK‐cell differentiation, selection, and activation.

### 2.3 Antiaging interventions on aged dogs

**FIGURE 2:**
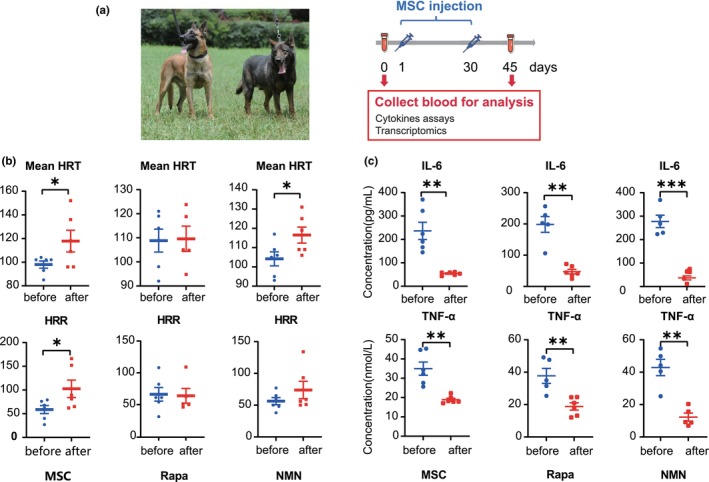
Effects of three treatments. (a) Schematic summary of the therapeutic strategy, mesenchymal stem cell (MSC) injection and sample collection. A dog was intravenously injected a dose of 5 × 10^7^ cells at 1 and 30 days. (b) Mean heart rate (mean HRT) and heart rate reserve (HRR) changes after MSC, nicotinamide mononucleotide (NMN) and rapamycin (Rapa) treatments. (c) Changes in cytokine levels after treatments. Concentrations of interleukin 6 (IL‐6) and tumor necrosis factor alpha (TNF‐α) in the plasma declined after three treatments. Data represent the mean ± SEM. ***p < 0.001, **p < 0.01, *p < 0.05 versus before treatment.

We recruited 17 retired police dogs for an antiaging study to evaluate how dogs respond to antiaging interventions. Belgian *Malinois* is a military working dog bred near the city of Malines in Belgium, and Kunming dogs were bred by crossing Chinese local native dogs with German shepherds (Figure 2a) (Wang, Cheng, et al., 2013). The average age of these dogs was 9 years, equivalent to 65 years for humans (Wang et al., 2020). The participating dogs were divided into three groups to further examine the effects of antiaging interventions, including allogeneic cMSCs, NMN, and rapamycin (Figure 2a and Table 1). As metabolism and immunity functions were most changed during aging, we measured heart rate and peripheral blood proinflammatory factors before and after treatments to evaluate the antiaging effects.

As shown in Figure 2b and Table S3, following MSC intervention, the mean heart rate (HRT) and heart rate reserve (HRR) showed significant increases of 19% and 74%, respectively; NMN intervention resulted in a significant increase of 11% in the mean HRT. However, neither was significant in the Rapa group. Comparatively, MSCs showed a better effect on improving cardiac function, suggesting that MSCs and NMN treatments can improve heart function.

Next, we tested the changes in the levels of interleukin‐6 (IL‐6) and tumor necrosis factor‐α (TNF‐α), two major proinflammatory cytokines that provide valuable information regarding the systemic inflammatory response and play important roles in aging (Rea et al., 2018). Both IL‐6 and TNF‐α were significantly decreased in all treatment groups (Figure 2c and Table S4), indicating that all three treatments effectively decreased systemic inflammation. Specifically, IL‐6 was reduced more than fourfold after treatment (236.36, 198.38, and 277.66 pg/mL) compared to before treatment (54.18, 47.31, and 36.67 pg/mL). TNF‐α was reduced approximately twofold after treatment (34.97, 37.71, and 42.86 pg/mL) compared to before treatment (18.88, 18.82, and 12.21 pg/mL). In summary, the above findings suggest that dogs respond well to antiaging treatments. Changes in cardiac function and inflammation after antiaging treatments provide support for the fact that changes in cardiac function and immune function are the major hallmarks during the aging of dogs.

### 2.4 Regulation of MSC treatment is enriched in the cell cycle and immune cell differentiation

**FIGURE 3:**
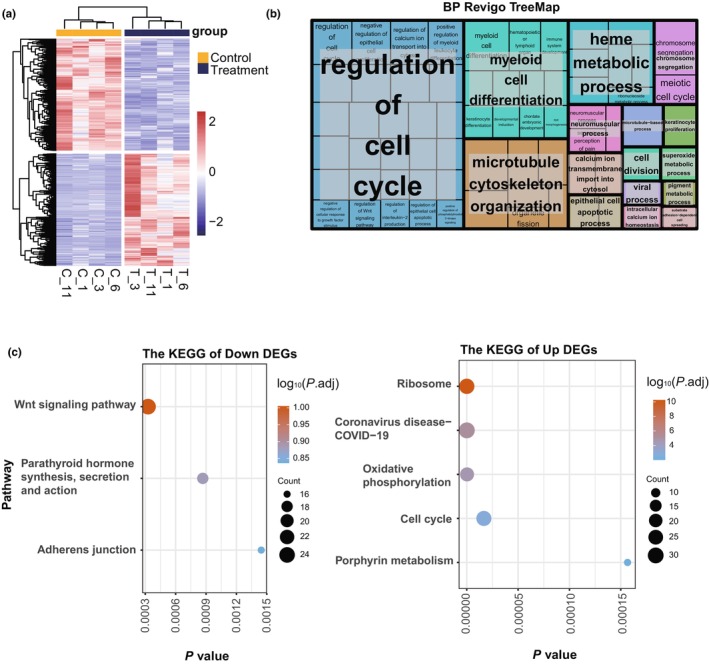
Gene Ontology (GO) and Kyoto Encyclopedia of Gene and Genomes (KEGG) enrichment of all differentially expressed genes (DEGs). (a) Heatmap of DEGs before and after mesenchymal stem cell treatment. (b) REVIGO tree map of biological process GO enrichment results. (c) KEGG enrichment analysis of downregulated DEGs and upregulated DEGs.

Since MSC treatment showed the best effect on improving cardiac function and anti‐inflammation, we performed whole‐blood RNA‐Seq on dogs treated with MSCs to identify potential mechanisms for its antiaging effect. The heatmap in Figure 3a shows the DEGs, and significant differences (Table S5) were noted in the clustering results between the two groups.

A total of 2349 genes were DEGs identified in the comparative analysis before versus after MSC treatment; 1176 genes were upregulated (50.1%), and 1173 genes were downregulated. Our results showed that the DEGs were mainly enriched in the regulation of the cell cycle, myeloid cell differentiation, microtubule cytoskeleton organization, and heme metabolic process by REVIGO analysis (Figure 3b), indicating that MSCs may play a major role in regulating the cell renewal system and immune cell differentiation. The downregulated DEGs were enriched in the Wnt signaling (cfa04310); parathyroid hormone synthesis, secretion and action (cfa04928); and adherens junction (cfa04520) pathways according to Kyoto Encyclopedia of Gene and Genomes (KEGG) enrichment analysis, and the upregulated DEGs were enriched in the ribosome (cfa03010), oxidative phosphorylation (cfa00190), cell cycle (cfa04110), and porphyrin metabolism (cfa00860) pathways (Figure 3c, Table S6). The effects of MSCs on the whole transcriptome were mainly enriched in cell metabolism, cell cycle, and immune cell differentiation, suggesting that MSCs may exert their antiaging effect by regulating cell senescence and immunity function in aged dogs.

### 2.5 Potential therapeutic target genes enriched in immunity

**FIGURE 4:**
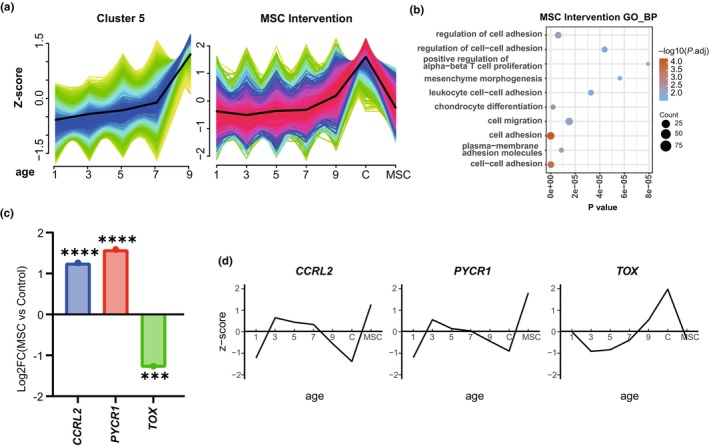
Potential therapeutic target genes. (a) Cluster 5 was upregulated abruptly only at ages 7–9 (left). After mesenchymal stem cell (MSC) intervention (MSC), the pattern was downregulated in the opposite direction compared with age 7 to 9 (right), and 9 was different from C (before injection), which may be due to individuals, as they are two different groups. (b) The biological process of GO enrichment analysis of intervention cluster. (c) Three potential therapeutic target genes after MSC treatment, log_2_‐fold change in gene expression, *p* value runs with the DESeq2 package in R using the Wald test, ***p < 0.001, ****p < 0.0001. (d) The pattern of these 3 key genes in baseline. Z score (y axis) was used to show relative changes in gene expression in age 1–9 and MSC intervention (x axis). C: before MSC intervention; MSC: after MSC intervention.

To observe the effects of MSC treatment on the baseline patterns, we combined the baseline and MSC treatment data (Figure S1b). The pattern increased abruptly in Cluster 5 (Figure 4a) at the aging phase and was fully regulated in the opposite direction. Genes in Cluster 5 were enriched in cell adhesion and migration; T‐cell proliferation; leukocyte cell–cell adhesion, and mesenchyme morphogenesis (Figure 4b). This may indicate that MSCs target the cell adhesion and adaptive immune system.

To explore potential therapeutic target genes, we screened the 61 A‐AGs that are regulated in the opposite direction after MSC treatment. The DEGs are summarized in Figure 4c,d and Table S7. Three potential age‐associated genes were screened in this study, two of which are implicated in immunity. Furthermore, *CCRL2* and *PYCR1* were downregulated with age since 3 years of age, and both were extremely significantly upregulated after MSC treatment, whereas *TOX* was downregulated. Intriguingly, *CCRL2* and *TOX* are closely related to immunity; *CCRL2* is critical for the recruitment of effector immune cells to the site of inflammation (Schioppa et al., 2020), and the protein encoded by *TOX* may function to regulate T‐cell development (Han et al., 2022). These data suggest that improvement of immune function may play an antiaging role in dogs and that dogs may be a good animal model for studying the role of inflammation in aging.

## 3 DISCUSSION

Dogs are a promising model of aging, especially in the study of multimorbidity (Hoffman et al., 2018; Ruple et al., 2022). We previously reported that humans and dogs have undergone convergent evolution, which supports the use of dogs as a good translational model for humans (Cao et al., 2021; Liu et al., 2021; G. D. Wang, Zhai, et al., 2013; Zhou et al., 2022). To gain more insights into aging in dogs, we profiled the transcriptomic patterns of dogs of different ages and identified 61 genes with dynamic changes with aging; the majority of genes were related to metabolism and immune function. Further intervention studies revealed that the declining immune function of aged dogs can be partially reversed.

The 61 A‐AGs could be considered predominant biomarkers for the aging process in dogs. These 61 one‐to‐one orthologous genes play roles in developmental programs, cell senescence, and immunosenescence in humans and dogs; notably, *PYCR1*, *ATF6B*, *TRPS1*, *MTHFR*, *SLC13A2*, *TOX*, *PLXNA4*, and *MEIS2* were found to be age‐associated in humans, and *GZMK* was found to be age‐associated in mice. A large proportion of them were related to metabolism and secondary genes related to immunity (up to 10/61), suggesting that metabolism and immune function changes may be the major hallmarks of dog aging. The metabolism genes are involved in cellular basal metabolism, such as the formation of the prereplicative complex (*LRWD1*), RNA splicing (*DDX39B*), RNA binding and tRNA binding (*TRMT1*), intra‐Golgi vesicle‐mediated transport (*COPG2*), transmembrane transport (*SLC16A12*), signaling *(MAGI3*), bile acid and bile salt transport (*SLC10A6*), bioactive sphingolipid metabolism (*SGPP1*), energy metabolism (*SLC13A2, ABCD2*), cell cycle (*PTPN13*), and cell differentiation (*FZD1*). Among immunity‐related genes, *CCRL2*, *DDX41*, *NUP85*, *CHIC2*, and *KLHL3* were found to be involved in innate immunity, whereas *KLRG1*, *GZMK*, *CD160*, *CD80*, and *TOX* were implicated in adaptive immunity. *PYCR1* is related to premature skin aging. *MEIS2* regulates endothelial to hematopoietic transition of human embryonic stem cells. *ATF6* is involved in organelle homeostasis and cellular aging. Consistent with human aging, cell senescence, and immunosenescence are the primary motivators.

We conducted the initial exploration with police dogs because they are in a relatively homogenous environment. Drugs such as rapamycin and NMN have the potential to delay aging, and MSCs may target the link between frailty and chronic inflammation to treat frailty (Schulman et al., 2018). Thus, we tested three treatments on aged dogs to explore biomarkers of response. Dogs showed significant declines in the function of all major organ systems with age, and we chose cardiac function to evaluate, which was studied previously with Rapa treatments (Kaeberlein et al., 2016; Urfer et al., 2017). MSC and NMN therapies substantially improve cardiac function and have shown the potential to become promising medical interventions to relieve symptoms of cardiac failure. All treatments substantially reduced important biomarkers of inflammation, as shown by IL‐6 and TNF‐α, the levels of which increase with age (Alexander et al., 2018). Briefly, our data suggest that classical antiaging treatments, especially MSCs, have a good response in reducing systematic inflammation in dogs.

Subsequent transcriptomic analysis showed that DEGs were mainly enriched in the cell cycle, immune cell differentiation, Wnt signaling, ribosome, and other cell metabolism pathways. Among them, the Wnt signaling pathways are involved in tumorigenesis, the cell cycle and other pathways (Zhan et al., 2017); increased ribosome pausing critically contributes to proteostasis impairment during translation elongation (Stein et al., 2022); and long‐term cell cycle arrest contributes to senescence. These pathways were significantly positively regulated, suggesting that antiaging therapies produce apparent effects on cell senescence.

Among the clusters in the aging baseline, Cluster 5 was abruptly unregulated at the aging phase, which was mainly enriched in cell adhesion and adaptive immune‐related GO terms, and it could be redressed by MSCs. Of the three potential therapeutic target genes we identified, *PYCR1* encodes an enzyme that catalyzes the NAD(P)H‐dependent conversion of pyrroline‐5‐carboxylate to proline, which is related to the maintenance of protein synthesis and cell proliferation (Zhu, Schworer, et al., 2021) and plays a physiologic role in the generation of NADP(+) in some cell types. It was included in the HAGR database as a human aging gene, and a study found that apoptosis and senescence were detected in juvenile *PCRY1* KO zebrafish (Liang et al., 2019). *CCRL2* and *TOX* were associated with immunosenescence because of the recruitment of geneogenous immunizing cells (Regan‐Komito et al., 2017) and T‐cell exhaustion (Han et al., 2022), which are biomarkers of immune dysregulation in seniors, and aged immune cells can promote systemic aging (Yousefzadeh et al., 2021). *CCRL2* depressed the recruitment of geneogenous immunizing cells to the sites of inflammation; it can increase the levels of chemerin, resulting in increased neutrophil and inflammatory monocyte recruitment, and it is selectively induced in immunostimulatory macrophages and functions to potentiate antitumor CD8 T‐cell responses (Yin et al., 2021). *CCRL2* is downregulated after 3 years of age and is significantly reversed by MSCs (*p* < 0.0001). The *TOX* subfamily comprises evolutionarily conserved DNA‐binding proteins involved in the control of cell apoptosis, growth, metastasis, DNA repair and so on (Yu & Li, 2015). T‐cell aging (Mittelbrunn & Kroemer, 2021) may be one of the principal manifestations of immunosenescence. T cells lose their quiescence and acquire a terminally differentiated stage, causing increasing autoinflammatory and autoimmune disease; *TOX* plays key roles in the development of CD4+ T lineages (Aliahmad & Kaye, 2008), which is associated with elevated circulating inflammatory cytokines, and inflammation interacts with CD4 T‐cell aging. Furthermore, *TOX* induces CD8 T cells to differentiate toward exhaustion, which leads to an increase in exhausted T cells. The naive: memory T‐cell ratio is one of the characteristics of immunosenescence (Cheng et al., 2021), which is also true for dogs (Greeley et al., 2001). These findings suggest that immunosenescence may be a predominant marker of aging in dogs, which may be an important target of MSCs, and MSCs may instrumentally slow immunosenescence.

In summary, we profiled the transcriptome of domestic dogs and revealed immune function decline as the major marker of dog aging. Antiaging interventions improved immunity and may reduce chronic inflammation in old dogs. These results suggest that domestic dogs may be a good model to study aging, especially anti‐inflammatory effects. However, it is worth noting that the limited sample size of our cohort may have caused a speculative conclusion. The present study recruited retired police dogs to keep the environment, lifestyle, and health as consistent as possible. The stringent criteria for recruiting individuals reduced the sample size and did not consider control group and sex effects. Future studies that include older and larger numbers of participants are needed to validate and extend the current findings, and it is also important to improve the quality of stem cell identification. Moreover, it will be important to complement single‐cell transcriptomes and ATACs to explore the molecular and cellular mechanisms of stem cells in immunosenescence. Given that dogs share the human environment and have undergone convergent evolution with humans, an international consortium such as the Dog Aging Project (Creevy et al., 2022) could facilitate discoveries on the biology of aging and improve the quality of life of old dogs.

## 4 MATERIALS AND METHODS

### 4.1 Animals and sample collection for baseline

A total of 30 healthy Belgian *Malinois* individuals were selected to construct baselines. They were 1, 3, 5, 7, and 9 years old with an average of 6 individuals per age, equivalent to human ages of 31, 49, 57, 62, 66, and 66+ years old, respectively. A unified screening and management program was used for these dogs. None of them were spayed or neutered. Male *Malinois* weighed 25–30 kg, while females weighed 22–27 kg. Male Kunming dogs weighed 35–43 kg, while females weighed 32–38 kg. None of them were overweight/underweight or pregnant. The dogs were fed at 8:30 am, and blood samples were collected 2–8 h after feeding.

### 4.2 RNA extraction and sequencing

Peripheral blood was collected for RNA sequencing at Novogene Co., Ltd. Total RNA was extracted from peripheral blood using TRIzol Reagent (Ambion, USA), and a total amount of 1 μg RNA per sample was used as input material for the RNA sample preparations. RNA integrity was assessed using the Bioanalyzer 2100 system (Agilent Technologies, CA, USA). Sequencing libraries were generated using the NEBNext® UltraTMRNA Library Prep Kit for Illumina® (NEB, USA) following the manufacturer's recommendations, and index codes were added to attribute sequences to each sample. The clustering of the index‐coded samples was performed on a cBot Cluster Generation System using TruSeq PE Cluster Kit v3‐cBot‐HS (Illumina) according to the manufacturer's instructions. After cluster generation, the library preparations were sequenced on an Illumina NovaSeq 6000 platform, and 150 bp paired‐end reads were generated.

### 4.3 Quality control of the data

To obtain clean data, fastp (v 0.12.4) was used to process the raw data. Reads containing adapters, poly‐N, and more than 50% low‐quality bases were filtered. Finally, we obtained a mean of 44.86 million reads per sample (Table S8). All downstream analyses were based on clean data.

### 4.4 Mapping and estimation of expression levels

Reads from mRNA‐Seq data were mapped to the Ensembl dog genome (CanFam3.1, Ensembl) using HISAT2 (v2.2.1). After mapping, raw gene counts (gene expression levels) were counted by FeatureCounts (v2.0.3), and Ensembl dog annotation was performed. Then, we normalized gene counts using the edgeR package, which scaled raw gene counts by library sizes (TMM method) and computed counts per million (CPM) values. Genes with total counts (in all samples) below 10 were filtered. This study was designed to use the domestic dog as an animal model, so we downloaded the list of orthologous genes among humans, dogs and mice from the OMA Orthology database. We finally obtained 12,358 orthologous genes with expression levels for the following analysis. As we sequenced whole‐blood samples, red blood cell genes needed to be removed. We referred to the human hemoglobin gene family provided by Harrington et al., which contains 12 human hemoglobin genes, 6 of which were on the human–dog orthologous gene list (OMA Orthology database), and 4 of which are present in the annotation file (http://ftp.ensembl.org/pub/release‐104/gtf/canis_lupus_familiaris/Canis_lupus_familiaris.CanFam3.1.104.gtf.gz). None of these genes were present in our gene set, so we retained all 12,358 orthologous genes for the downstream analysis. The aging and treatment transcriptomic data were obtained in two separate batches, so we used ComBat‐seq to remove the batch effects. We specified “sex,” “age,” “breed,” and “group” as biological covariates, and these signals were preserved in the adjusted data (Table S9). We specified the batch variable for adjusting the batch effects (Figure S2). When we jointly analyzed these two sets of data, we used the count data that removed the batch effects.

### 4.5 PCA and correlation analysis

We evaluated the quality of the sequenced libraries using principal component analysis (PCA) and correlation analysis using R. FactoMineR (v2.4) was used to perform PCA. The results are provided in Figures S2a,b.

### 4.6 Clustering for all genes

To create an overview of the expression pattern of all genes across all ages, we used *Mfuzz* (v2.52.0) to cluster all the genes and draw the expression pattern. *Mfuzz* is an R package that uses soft clustering; soft clustering is more noise robust, and a priori prefiltering of genes can be avoided. It can also define a global clustering structure. For estimation of the optimized number of clusters, we use Dmin() to calculate the minimum centroid distance for a range of cluster numbers and cselection() to repeat soft clustering for detection of empty clusters. We found that the optimized number of clusters is 8 and 6, and we set the “center = T” parameter to draw a line for the cluster center.

### 4.7 GO analysis and KEGG enrichment

To assess the biological processes and pathways in which the genes of interest were significantly enriched, we used the clusterProfiler R package to conduct GO and KEGG pathway analyses. The background gene set included all the orthologous genes in our analysis (*N* = 12,358), and the enrichment database was org.Cf.eg.db (V3.5.0). We also used REVIGO to identify a representative subset of the GO terms; this web‐based tool summarizes long, unintelligible lists of GO terms using a simple clustering algorithm.

### 4.8 Age‐associated genes

We used two methods, as shown in Figure S1e and Table S10, ImpulseDE2 (Fischer et al., 2019) v1.8.0, and maSigPro v1.64.0, to identify A‐AGs (*p*adj <0.05). After comparison, it was found that all the genes identified by ImpulseDE2 were also identified by maSigPro. maSigPro is an R package designed for transcriptomic time courses that includes age as a continuous variable to compute a regression fit for each gene. Then, it computes the *p* value associated with the F‐statistic of the model, which is used to select significant genes. By default, maSigPro corrects this *p*‐value for multiple comparisons by using the Benjamini–Hochberg (B‐H) false discovery rate (FDR) procedure. The significant genes showed expression changes with aging. Therefore, for normal dog blood samples, we used maSigPro (v1.64.0) to identify the genes with dynamic expression profiles. We used the count matrix normalized from EdgeR as input, degree = 3 (polynomial). We considered genes as A‐AGs when the p.adjusted of p.vector() was less than 0.05, which means that these genes were significantly correlated with aging. Then, we used Mfuzz (v2.52.0) to cluster these genes according to their expression profiles.

### 4.9 DEG analysis

DESeq2 (v1.32.0) (Love et al., 2014) packages were used to identify DEGs between control and treated dog samples (two biological replicates per condition), and genes with adjusted *p*‐value <0.05 and |log2(fold change)| >1.0 were defined as significantly differentially expressed. To consider the effects of sex and individual, we also included sex and individual as the covariables, together with the treatment variable, in the design formula: design = ~sex + individual + treatment (the function used the last variable in the formula for building results).

### 4.10 Primary culture and identification of cMSCs from umbilical cord

Canine MSCs (cMSCs) were isolated from the umbilical cord (UC), which was obtained by caesarean operation in beagles at Beijing Sincgene Biotechnology Co., Ltd. After the tissues were obtained, they were sterilized by immersion in 75% alcohol for 5 s and washed three times in PBS containing 2% penicillin–streptomycin (P‐S, 10,000 U/mL, Thermo Fisher, USA). After that, the tissues were placed in a 50 mL centrifuge tube containing approximately 20 mL of MACS tissue storage solution (Miltenyi Biotec, Germany), sealed with sealing film, and transported at 4°C for approximately 2 days.

The following aseptic procedure was performed in a biological safety cabinet: the tissue was washed in PBS containing 2%, 5%, and 10% P‐S; blood and vessels were removed; and the tissue was cut into pieces, which were then processed enzymatically with mechanical dissociation. The tissues were then minced and digested in sterile filtered mixed digestive solution (approximately 10 times the volume of the tissue), prepared by mixing 0.2% collagenase IV (Gibco, USA), 0.25% trypsin (Thermo, USA) and 0.1% hyaluronidase (Sigma, USA) at a volume ratio of 2:1:1 in Hanks' balanced salt solution (with Ca^2+^ and Mg^2+^) according to our patented proprietary technology, working under sterile conditions, after 30–60 min (adjusting the time according to the size of the tissue) of incubation in a water bath at 37°C, and shaking several times during incubation.

Subsequently, the suspension was centrifuged at 1000 rpm for 5 min, repeated twice. The resulting pellet was resuspended in maintenance medium containing Dulbecco's modified eagle medium (DMEM) basic with 1 g/L D‐Glucose (Gibco, USA), 10% fetal bovine serum (FBS, Gibco, USA), 2% P‐S, 1% 100 mM sodium pyruvate (Gibco, USA), 1% 200 mM L‐glutamine (Gibco, USA) and 1% 0.1 mM MEM NEAA (Gibco, USA). All cultures were maintained at 37°C and 5% CO_2_ in a humidified incubator. The medium was discharged, and fresh medium was added when the single cells were clearly attached. Following this step, the culture medium was changed every 72–96 h until the cells reached 80%–90% confluence.

The growth curve of the MSCs at passage 1 (P1) is shown in Figure S3a. cMSCs maximized proliferation on the seventh day. During the primary culture, cMSCs adhered to the plastic dishes in a scattered manner, exhibited similar morphology to each other, and appeared plastic‐adherent and fibroblast‐like.

The MSCs maintained their fibroblast‐like morphology and were frozen at P3. The resulting pellet was resuspended in 1 mL frozen stock solution with 10% DMSO, 20% FBS, 1% P‐S, and DMEM Basic. Twenty microliters of the resuspension were mixed with 20 μL AO/PI dyes. Approximately 5 min later, the cells were counted with a fluorescence cell analyzer (Countstar Biotech, ALIT Life Science, China), and the cell concentration and diameter were recorded. The increase in cell volume was an initial assessment of cellular senescence. The remaining cell resuspension was transferred to 2 mL cryogenic vials (Corning, USA) and placed in a freezing container (Corning, USA). The samples were frozen for a minimum of 4 h before being transferred to liquid nitrogen for long‐term storage.

The cryogenic vials of the frozen cells were removed from liquid nitrogen storage and immediately placed into a 37°C water bath. The cells were quickly thawed (<1 min) by gently swirling the vial in the 37°C water bath until there was just a small bit of ice left in the vial. After centrifugation and suspension, the cells were gently resuspended in the medium and transferred into a 100 mm × 20 mm dish (Corning, USA) and placed into a CO_2_ incubator (Thermo, USA).

### 4.11 Cell differentiation

The MSCs were tested for the capability of trilineage differentiation into adipocytes, osteocytes, and chondrocytes with commercial differentiation kits (Cyagen, China) according to the manufacturer's manual. Briefly, for adipocyte differentiation, MSCs were plated in 6‐well plates at 1 × 10^5^ cells/well, and the adipogenic differentiation media were changed every other day for 21 days. The cells were fixed with 4% paraformaldehyde (PFA) for 30 min at 4°C. Then, the cells were assessed by staining them with a 0.5% Oil Red O solution. For osteogenic differentiation, MSC administration, osteogenic differentiation medium change, culture days and fixation were the same as those for adipogenic differentiation, and the mineralization of differentiated cells was assessed by staining with Alizarin red. For chondrogenic differentiation, 1 × 10^5^ MSCs were centrifuged at 1000 rpm for 5 min to form the cell pellets, and then, the cell pellets were cultured in chondrogenic differentiation medium for 21 days, fixed in 4% PFA and embedded in OCT (Sakura, Japan) for slicing with 5‐micron‐thick slices into a cryostat (Leica, Germany). Afterward, the sections were stained with toluidine blue.

The morphology and trilineage differentiation potential of these cMSCs were examined at P4. None of the cells were contaminated with bacteria, fungi, or mycoplasma. The cMSCs could differentiate into adipocytes, osteocytes, and chondrocytes (Figure S3b) and expressed the markers (Zhan et al., 2019) shown in Table S11. Overall, cMSCs derived from the umbilical cord met the criteria for cMSCs in this study.

### 4.12 Therapeutic regimen and sample collection

A total of 17 healthy Belgian *Malinois* and Cynopterus sphinx were recruited for cell and drug therapy; their average weight was 40 kg, male Kunming dogs weighed 35–43 kg, while females weighed 32–38 kg and their average age was 9 years old, equivalent to 65 in humans. They were divided into three groups and treated with allogeneic MSCs, NMN and rapamycin. A group was i.v. infected at a dosage of 5 × 10^7^ cells/dog on Days 1 and 30. The other two groups received dosages of 2.5 g/dog NMN (Mills et al., 2016) and 3 mg/dog Rapa every 2 days by oral administration. Peripheral blood samples were collected before the first injection and 15 days after the second injection, 2 h after feeding. None of them were sterilized and, overweight/underweight.

Only four samples in the MSC group passed the quality test, and they were collected for RNA‐Seq analysis. The technical routes are shown in Figure S4.

### 4.13 Heart rate and enzyme‐linked immunosorbent assays

A Polar heart rate monitor (model: Polar M430) was used to measure each dog's heart rate after running on a treadmill for 15 min at a pace of 4–10 km/h. During the run, the real‐time heart rate was continuously monitored. Whole blood was collected in EDTA anticoagulant tubes and separated by centrifugation at 2364 rpm for 20 min at 4°C to recover plasma. The plasma was immediately frozen at −80°C and stored until sufficient numbers of samples were collected to complete replicate analysis on an enzyme‐linked immunosorbent assay (ELISA) plate. All samples were assayed in duplicate. ELISAs were performed using canine IL‐6 and TNF‐α assay plates (J&L Biological, China) according to the manufacturer's instructions. The OD ratio was determined using BioTek EPOCH2 (Agilent, USA).

### 4.14 Statistical analysis

Statistical analysis was performed using GraphPad Prism software (v9.4.0). All data are presented as the mean ± standard error of the mean (SEM). A Shapiro–Wilk test was used to verify the normality of the data. An unpaired t test with Welch's correction was used to detect differences between the control and treatment groups. A probability (*p*) value of <0.05 was considered statistically significant. All experiments were performed using at least three independent samples.

## AUTHOR CONTRIBUTIONS

G‐D.W. and Y.H.H. conceived and designed the study. M.Z., T.Z., and Z.Y.L. worked on the experiments and analyzed the data. M.Z., T.Z., L.W., P. N. S., G.‐D.W., and Y.H.H. drafted the manuscript. S.R.Z. and G.M.L. helped with the culture and identified the MSCs. The rest of the authors helped to recruited dogs and collect samples. All the authors have read and approved the final manuscript.

## FUNDING INFORMATION

This work was supported by the National Key R&D Program of China (2019YFA0707101, 2023YFC3603300), STI2030‐Major Projects (2021ZD0203900), Spring City Plan: The High‐level Talent Promotion and Training Project of Kunming (2022SCP001), Yunnan Fundamental Research Projects (202201AV070011, 202201AS070038 and 202305AH340006), National Natural Science Foundation of China (82171558), and State Key Laboratory for Conservation and Utilization of Bio‐Resources in Yunnan, Yunnan University (2021KF006). G.‐D.W. is supported by the Youth Innovation Promotion Association of CAS. Y. H. H. is supported by the Pioneer Hundred Talents Program of the Chinese Academy of Sciences and the Yunnan Revitalization Talent Support Program Young Talent Project.

## CONFLICT OF INTEREST STATEMENT

The authors declare that they have no competing interests.

## Supporting information


Figure S1.

Figure S2.

Figure S3.

Figure S4.

Table S1.

Table S2.

Table S3.

Table S4.

Table S5.

Table S6.

Table S7.

Table S8.

Table S9.

Table S10.

Table S11.

Captions.

## Data Availability

The data used in this study are available in the Genome Sequence Archive in the BIG Data Center, Beijing Institute of Genomics (BIG), Chinese Academy of Sciences, and can be accessed with PRJCA016985.

## References

[acel14127-bib-0001] Alexander, J. E. , Colyer, A. , Haydock, R. M. , Hayek, M. G. , & Park, J. (2018). Understanding how dogs age: Longitudinal analysis of markers of inflammation, immune function, and oxidative stress. The Journals of Gerontology. Series A, Biological Sciences and Medical Sciences, 73(6), 720–728. 10.1093/gerona/glx182 29126143

[acel14127-bib-0002] Aliahmad, P. , & Kaye, J. (2008). Development of all CD4 T lineages requires nuclear factor TOX. The Journal of Experimental Medicine, 205(1), 245–256. 10.1084/jem.20071944 18195075 PMC2234360

[acel14127-bib-0003] Barnett, B. G. , Wesselowski, S. R. , Gordon, S. G. , Saunders, A. B. , Promislow, D. E. L. , Schwartz, S. M. , Chou, L. , Evans, J. B. , Kaeberlein, M. , & Creevy, K. E. (2023). A masked, placebo‐controlled, randomized clinical trial evaluating safety and the effect on cardiac function of low‐dose rapamycin in 17 healthy client‐owned dogs. Frontiers in Veterinary Science, 10, 1168711. 10.3389/fvets.2023.1168711 37275618 PMC10233048

[acel14127-bib-0004] Cao, X. , Liu, W. P. , Cheng, L. G. , Li, H. J. , Wu, H. , Liu, Y. H. , Chen, C. , Xiao, X. , Li, M. , Wang, G. D. , & Zhang, Y. P. (2021). Whole genome analyses reveal significant convergence in obsessive‐compulsive disorder between humans and dogs. Science Bulletin, 66(2), 187–196. 10.1016/j.scib.2020.09.021 36654227

[acel14127-bib-0005] Cardoso‐Moreira, M. , Halbert, J. , Valloton, D. , Velten, B. , Chen, C. , Shao, Y. , Liechti, A. , Ascencao, K. , Rummel, C. , Ovchinnikova, S. , Mazin, P. V. , Xenarios, I. , Harshman, K. , Mort, M. , Cooper, D. N. , Sandi, C. , Soares, M. J. , Ferreira, P. G. , Afonso, S. , … Kaessmann, H. (2019). Gene expression across mammalian organ development. Nature, 571(7766), 505–509. 10.1038/s41586-019-1338-5 31243369 PMC6658352

[acel14127-bib-0006] Cesari, M. , Prince, M. , Thiyagarajan, J. A. , De Carvalho, I. A. , Bernabei, R. , Chan, P. , Gutierrez‐Robledo, L. M. , Michel, J. P. , Morley, J. E. , Ong, P. , Rodriguez Manas, L. , Sinclair, A. , Won, C. W. , Beard, J. , & Vellas, B. (2016). Frailty: An emerging public health priority. Journal of the American Medical Directors Association, 17(3), 188–192. 10.1016/j.jamda.2015.12.016 26805753

[acel14127-bib-0007] Cheng, Y. , Shao, Z. , Chen, L. , Zheng, Q. , Zhang, Q. , Ding, W. , Zhang, M. , Yu, Q. , & Gao, D. (2021). Role, function and regulation of the thymocyte selection‐associated high mobility group box protein in CD8(+) T cell exhaustion. Immunology Letters, 229, 1–7. 10.1016/j.imlet.2020.11.004 33186634

[acel14127-bib-0008] Creevy, K. E. , Akey, J. M. , Kaeberlein, M. , Promislow, D. E. L. , & Dog Aging Project Consortium . (2022). An open science study of ageing in companion dogs. Nature, 602(7895), 51–57. 10.1038/s41586-021-04282-9 35110758 PMC8940555

[acel14127-bib-0009] Fischer, D. S. , Theis, F. J. , & Yosef, N. (2019). ImpulseDE2: Differential expression analysis of longitudinal count data sets.

[acel14127-bib-0010] Greeley, E. H. , Ballam, J. M. , Harrison, J. M. , Kealy, R. D. , Lawler, D. F. , & Segre, M. (2001). The influence of age and gender on the immune system: A longitudinal study in Labrador retriever dogs. Veterinary Immunology and Immunopathology, 82(1), 57–71. 10.1016/S0165-2427(01)00336-1 11557294

[acel14127-bib-0011] Han, J. , Wan, M. , Ma, Z. , & He, P. (2022). The TOX subfamily: All‐round players in the immune system. Clinical and Experimental Immunology, 208(3), 268–280. 10.1093/cei/uxac037 35485425 PMC9226143

[acel14127-bib-0012] Hoffman, J. M. , Creevy, K. E. , Franks, A. , O'Neill, D. G. , & Promislow, D. E. L. (2018). The companion dog as a model for human aging and mortality. Aging Cell, 17(3), e12737. 10.1111/acel.12737 29457329 PMC5946068

[acel14127-bib-0013] Holtze, S. , Gorshkova, E. , Braude, S. , Cellerino, A. , Dammann, P. , Hildebrandt, T. B. , Hoeflich, A. , Hoffmann, S. , Koch, P. , Terzibasi Tozzini, E. , Skulachev, M. , Skulachev, V. P. , & Sahm, A. (2021). Alternative animal models of aging research. Frontiers in Molecular Biosciences, 8, 660959. 10.3389/fmolb.2021.660959 34079817 PMC8166319

[acel14127-bib-0014] Kaeberlein, M. , Creevy, K. E. , & Promislow, D. E. (2016). The dog aging project: Translational geroscience in companion animals. Mammalian Genome, 27(7–8), 279–288. 10.1007/s00335-016-9638-7 27143112 PMC4936929

[acel14127-bib-0015] Kumar, L. , & Futschik, M. E. (2007). Mfuzz: A software package for soft clustering of microarray data. Bioinformation, 2(1), 5–7. 10.6026/97320630002005 18084642 PMC2139991

[acel14127-bib-0016] Liang, S. T. , Audira, G. , Juniardi, S. , Chen, J. R. , Lai, Y. H. , Du, Z. C. , Lin, D. S. , & Hsiao, C. D. (2019). Zebrafish carrying pycr1 gene deficiency display aging and multiple behavioral abnormalities. Cell, 8(5), 453. 10.3390/cells8050453 PMC6562453 31091804

[acel14127-bib-0017] Liu, Y. H. , Wang, L. , Zhang, Z. , Otecko, N. O. , Khederzadeh, S. , Dai, Y. , Liang, B. , Wang, G. D. , & Zhang, Y. P. (2021). Whole‐genome sequencing reveals lactase persistence adaptation in European dogs. Molecular Biology and Evolution, 38(11), 4884–4890. 10.1093/molbev/msab214 34289055 PMC8557436

[acel14127-bib-0018] Lopez‐Otin, C. , Blasco, M. A. , Partridge, L. , Serrano, M. , & Kroemer, G. (2023). Hallmarks of aging: An expanding universe. Cell, 186(2), 243–278. 10.1016/j.cell.2022.11.001 36599349

[acel14127-bib-0019] Love, M. I. , Huber, W. , & Anders, S. (2014). Moderated estimation of fold change and dispersion for RNA‐seq data with DESeq2. Genome Biology, 15(12), 550. 10.1186/s13059-014-0550-8 25516281 PMC4302049

[acel14127-bib-0020] Mills, K. F. , Yoshida, S. , Stein, L. R. , Grozio, A. , Kubota, S. , Sasaki, Y. , Redpath, P. , Migaud, M. E. , Apte, R. S. , Uchida, K. , Yoshino, J. , & Imai, S. I. (2016). Long‐term administration of nicotinamide mononucleotide mitigates age‐associated physiological decline in mice. Cell Metabolism, 24(6), 795–806. 10.1016/j.cmet.2016.09.013 28068222 PMC5668137

[acel14127-bib-0021] Mittelbrunn, M. , & Kroemer, G. (2021). Hallmarks of T cell aging. Nature Immunology, 22(6), 687–698. 10.1038/s41590-021-00927-z 33986548

[acel14127-bib-0022] Neff, F. , Flores‐Dominguez, D. , Ryan, D. P. , Horsch, M. , Schroder, S. , Adler, T. , Afonso, L. C. , Aguilar‐Pimentel, J. A. , Becker, L. , Garrett, L. , Hans, W. , Hettich, M. M. , Holtmeier, R. , Holter, S. M. , Moreth, K. , Prehn, C. , Puk, O. , Racz, I. , Rathkolb, B. , … Ehninger, D. (2013). Rapamycin extends murine lifespan but has limited effects on aging. The Journal of Clinical Investigation, 123(8), 3272–3291. 10.1172/JCI67674 23863708 PMC3726163

[acel14127-bib-0023] Nueda, A. C. M. J. (2021). maSigPro: Significant gene expression profile differences in time course gene expression data .

[acel14127-bib-0024] Palliyaguru, D. L. , Shiroma, E. J. , Nam, J. K. , Duregon, E. , Teixeira, V. L. , C. , Price, N. L. , Bernier, M. , Camandola, S. , Vaughan, K. L. , Colman, R. J. , Deighan, A. , Korstanje, R. , Peters, L. L. , Dickinson, S. L. , Ejima, K. , Simonsick, E. M. , Launer, L. J. , Chia, C. W. , … de Cabo, R. (2021). Fasting blood glucose as a predictor of mortality: Lost in translation. Cell Metabolism, 33(11), 2189–2200.e3. 10.1016/j.cmet.2021.08.013 34508697 PMC9115768

[acel14127-bib-0025] Rea, I. M. , Gibson, D. S. , McGilligan, V. , McNerlan, S. E. , Alexander, H. D. , & Ross, O. A. (2018). Age and age‐related diseases: Role of inflammation triggers and cytokines. Frontiers in Immunology, 9, 586. 10.3389/fimmu.2018.00586 29686666 PMC5900450

[acel14127-bib-0026] Regan‐Komito, D. , Valaris, S. , Kapellos, T. S. , Recio, C. , Taylor, L. , Greaves, D. R. , & Iqbal, A. J. (2017). Absence of the non‐signalling chemerin receptor CCRL2 exacerbates acute inflammatory responses in vivo. Frontiers in Immunology, 8, 1621. 10.3389/fimmu.2017.01621 29209334 PMC5702352

[acel14127-bib-0027] Ruple, A. , MacLean, E. , Snyder‐Mackler, N. , Creevy, K. E. , & Promislow, D. (2022). Dog models of aging. Annual Review of Animal Biosciences, 10, 419–439. 10.1146/annurev-animal-051021-080937 34699257 PMC8962603

[acel14127-bib-0028] Schioppa, T. , Sozio, F. , Barbazza, I. , Scutera, S. , Bosisio, D. , Sozzani, S. , & Del Prete, A. (2020). Molecular basis for CCRL2 regulation of leukocyte migration. Frontiers in Cell and Developmental Biology, 8, 615031. 10.3389/fcell.2020.615031 33363177 PMC7758318

[acel14127-bib-0029] Schulman, I. H. , Balkan, W. , & Hare, J. M. (2018). Mesenchymal stem cell therapy for aging frailty. Frontiers in Nutrition, 5, 108. 10.3389/fnut.2018.00108 30498696 PMC6249304

[acel14127-bib-0030] Stein, K. C. , Morales‐Polanco, F. , van der Lienden, J. , Rainbolt, T. K. , & Frydman, J. (2022). Ageing exacerbates ribosome pausing to disrupt cotranslational proteostasis. Nature, 601(7894), 637–642. 10.1038/s41586-021-04295-4 35046576 PMC8918044

[acel14127-bib-0031] Urfer, S. R. , Kaeberlein, T. L. , Mailheau, S. , Bergman, P. J. , Creevy, K. E. , Promislow, D. E. L. , & Kaeberlein, M. (2017). A randomized controlled trial to establish effects of short‐term rapamycin treatment in 24 middle‐aged companion dogs. Geroscience, 39(2), 117–127. 10.1007/s11357-017-9972-z 28374166 PMC5411365

[acel14127-bib-0032] Wang, G. D. , Cheng, L. G. , Fan, R. X. , Irwin, D. M. , Tang, S. S. , Peng, J. G. , & Zhang, Y. P. (2013). Signature of balancing selection at the MC1R gene in Kunming dog populations. PLoS One, 8(2), e55469. 10.1371/journal.pone.0055469 23424634 PMC3570536

[acel14127-bib-0033] Wang, G. D. , Zhai, W. , Yang, H. C. , Fan, R. X. , Cao, X. , Zhong, L. , Wang, L. , Liu, F. , Wu, H. , Cheng, L. G. , Poyarkov, A. D. , Poyarkov, N. A., Jr. , Tang, S. S. , Zhao, W. M. , Gao, Y. , Lv, X. M. , Irwin, D. M. , Savolainen, P. , Wu, C. I. , & Zhang, Y. P. (2013). The genomics of selection in dogs and the parallel evolution between dogs and humans. Nature Communications, 4, 1860. 10.1038/ncomms2814 23673645

[acel14127-bib-0034] Wang, T. , Ma, J. , Hogan, A. N. , Fong, S. , Licon, K. , Tsui, B. , Kreisberg, J. F. , Adams, P. D. , Carvunis, A. R. , Bannasch, D. L. , Ostrander, E. A. , & Ideker, T. (2020). Quantitative translation of dog‐to‐human aging by conserved remodeling of the DNA methylome. Cell Systems, 11(2), 176–185.e6. 10.1016/j.cels.2020.06.006 32619550 PMC7484147

[acel14127-bib-0035] Yin, W. , Li, Y. , Song, Y. , Zhang, J. , Wu, C. , Chen, Y. , Miao, Y. , Lin, C. , Lin, Y. , Yan, D. , Chen, J. , & He, R. (2021). CCRL2 promotes antitumor T‐cell immunity via amplifying TLR4‐mediated immunostimulatory macrophage activation. Proceedings of the National Academy of Sciences of the United States of America, 118(16), e2024171118. 10.1073/pnas.2024171118 33846258 PMC8072249

[acel14127-bib-0036] Yousefzadeh, M. J. , Flores, R. R. , Zhu, Y. , Schmiechen, Z. C. , Brooks, R. W. , Trussoni, C. E. , Cui, Y. , Angelini, L. , Lee, K. A. , McGowan, S. J. , Burrack, A. L. , Wang, D. , Dong, Q. , Lu, A. , Sano, T. , O'Kelly, R. D. , McGuckian, C. A. , Kato, J. I. , Bank, M. P. , … Niedernhofer, L. J. (2021). An aged immune system drives senescence and ageing of solid organs. Nature, 594(7861), 100–105. 10.1038/s41586-021-03547-7 33981041 PMC8684299

[acel14127-bib-0037] Yu, X. , & Li, Z. (2015). TOX gene: A novel target for human cancer gene therapy. American Journal of Cancer Research, 5(12), 3516–3524. https://www.ncbi.nlm.nih.gov/pubmed/26885442 26885442 PMC4731627

[acel14127-bib-0038] Zhan, T. , Rindtorff, N. , & Boutros, M. (2017). Wnt signaling in cancer. Oncogene, 36(11), 1461–1473. 10.1038/onc.2016.304 27617575 PMC5357762

[acel14127-bib-0039] Zhan, X. S. , El‐Ashram, S. , Luo, D. Z. , Luo, H. N. , Wang, B. Y. , Chen, S. F. , Bai, Y. S. , Chen, Z. S. , Liu, C. Y. , & Ji, H. Q. (2019). A comparative study of biological characteristics and transcriptome profiles of mesenchymal stem cells from different canine tissues. International Journal of Molecular Sciences, 20(6), 1485. 10.3390/ijms20061485 30934541 PMC6471769

[acel14127-bib-0040] Zhou, Q. J. , Liu, X. , Zhang, L. , Wang, R. , Yin, T. , Li, X. , Li, G. , He, Y. , Ding, Z. , Ma, P. , Wang, S. Z. , Mao, B. , Zhang, S. , & Wang, G. D. (2022). A single‐nucleus transcriptomic atlas of the dog hippocampus reveals the potential relationship between specific cell types and domestication. National Science Review, 9(11), nwac147. 10.1093/nsr/nwac147 36569494 PMC9772819

[acel14127-bib-0041] Zhu, J. , Schworer, S. , Berisa, M. , Kyung, Y. J. , Ryu, K. W. , Yi, J. , Jiang, X. , Cross, J. R. , & Thompson, C. B. (2021). Mitochondrial NADP(H) generation is essential for proline biosynthesis. Science, 372(6545), 968–972. 10.1126/science.abd5491 33888598 PMC8241437

[acel14127-bib-0042] Zhu, Y. , Ge, J. , Huang, C. , Liu, H. , & Jiang, H. (2021). Application of mesenchymal stem cell therapy for aging frailty: From mechanisms to therapeutics. Theranostics, 11(12), 5675–5685. 10.7150/thno.46436 33897874 PMC8058725

