# Supplementary figures and images for: Transcriptomic and intervention evidence reveals domestic dogs as a promising model for anti‐inflammatory investigation
Source: Aging Cell. 2024 Mar 1;23(5):e14127. doi: 10.1111/acel.14127 (PMC11113267; doi:10.1111/acel.14127)

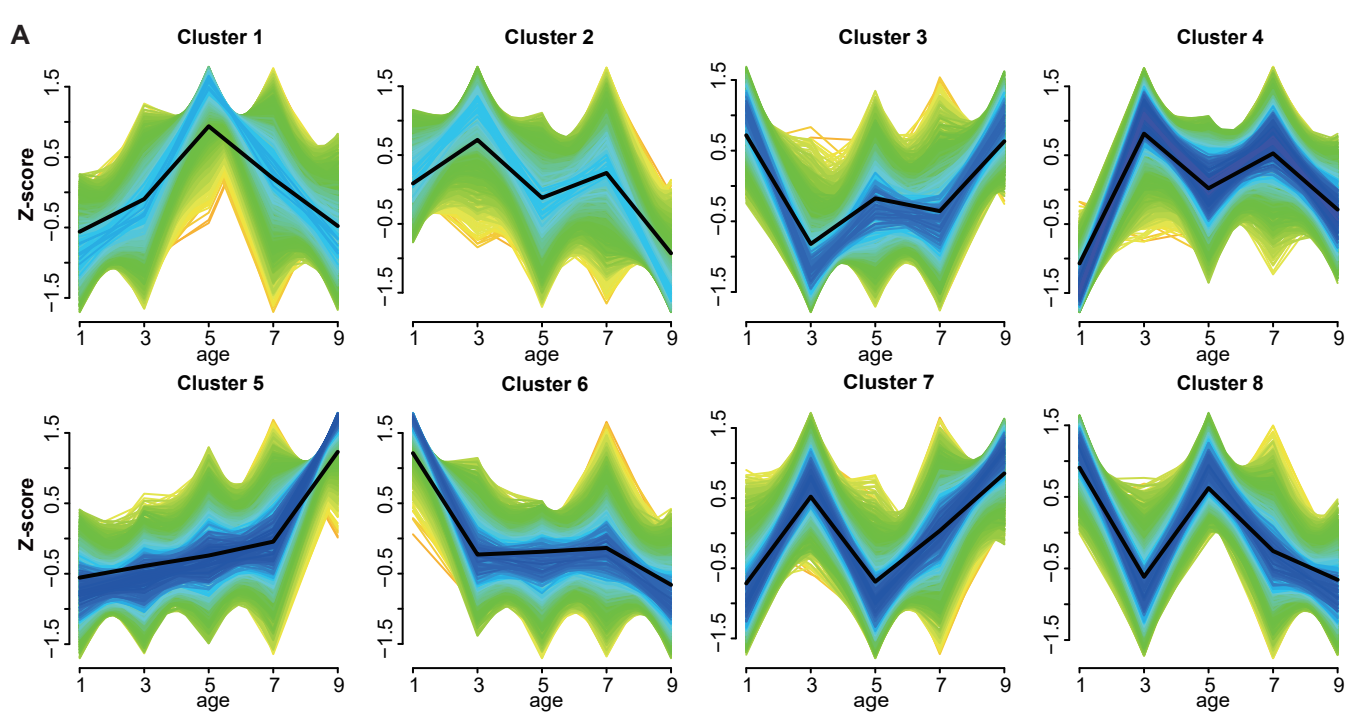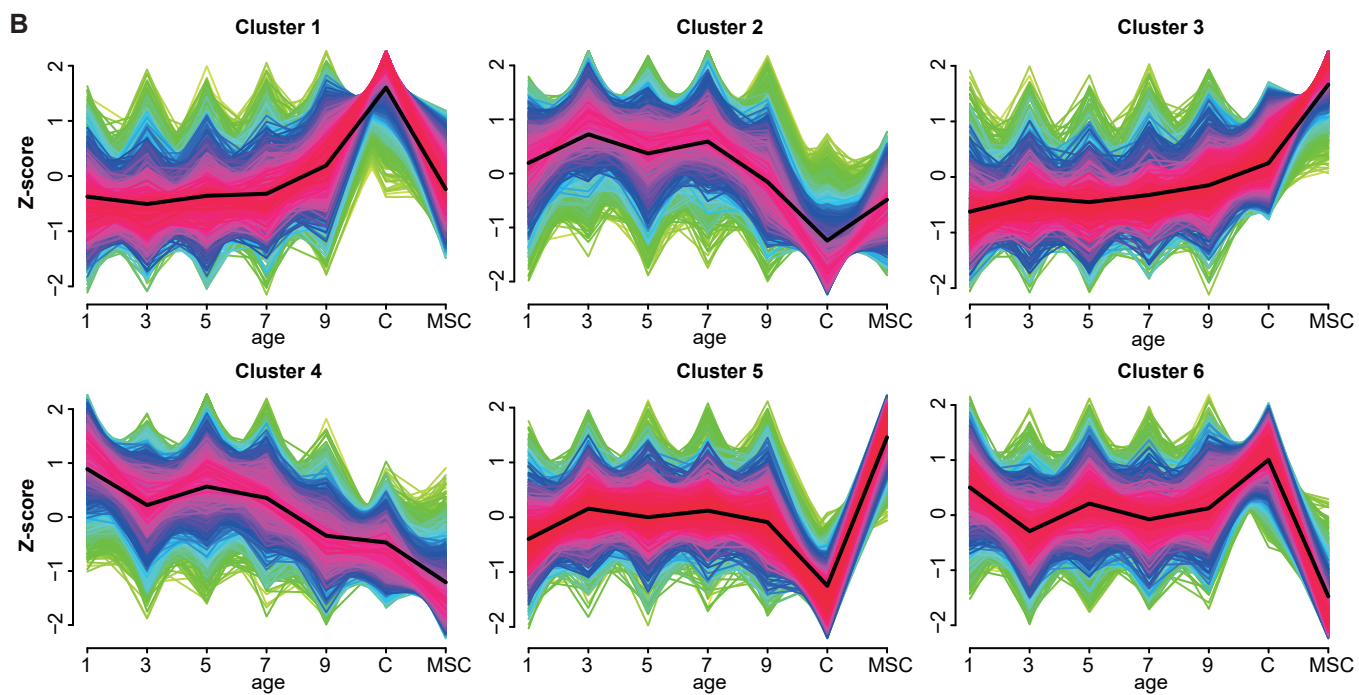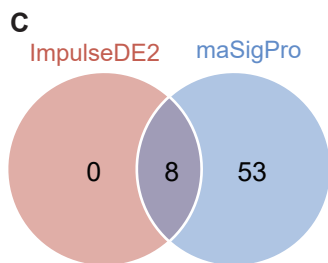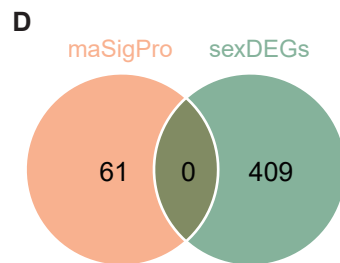

Supplement: Supplementary file 1 — Figure S1. [file ACEL-23-e14127-s009.pdf]

**A** Individuals – PCA

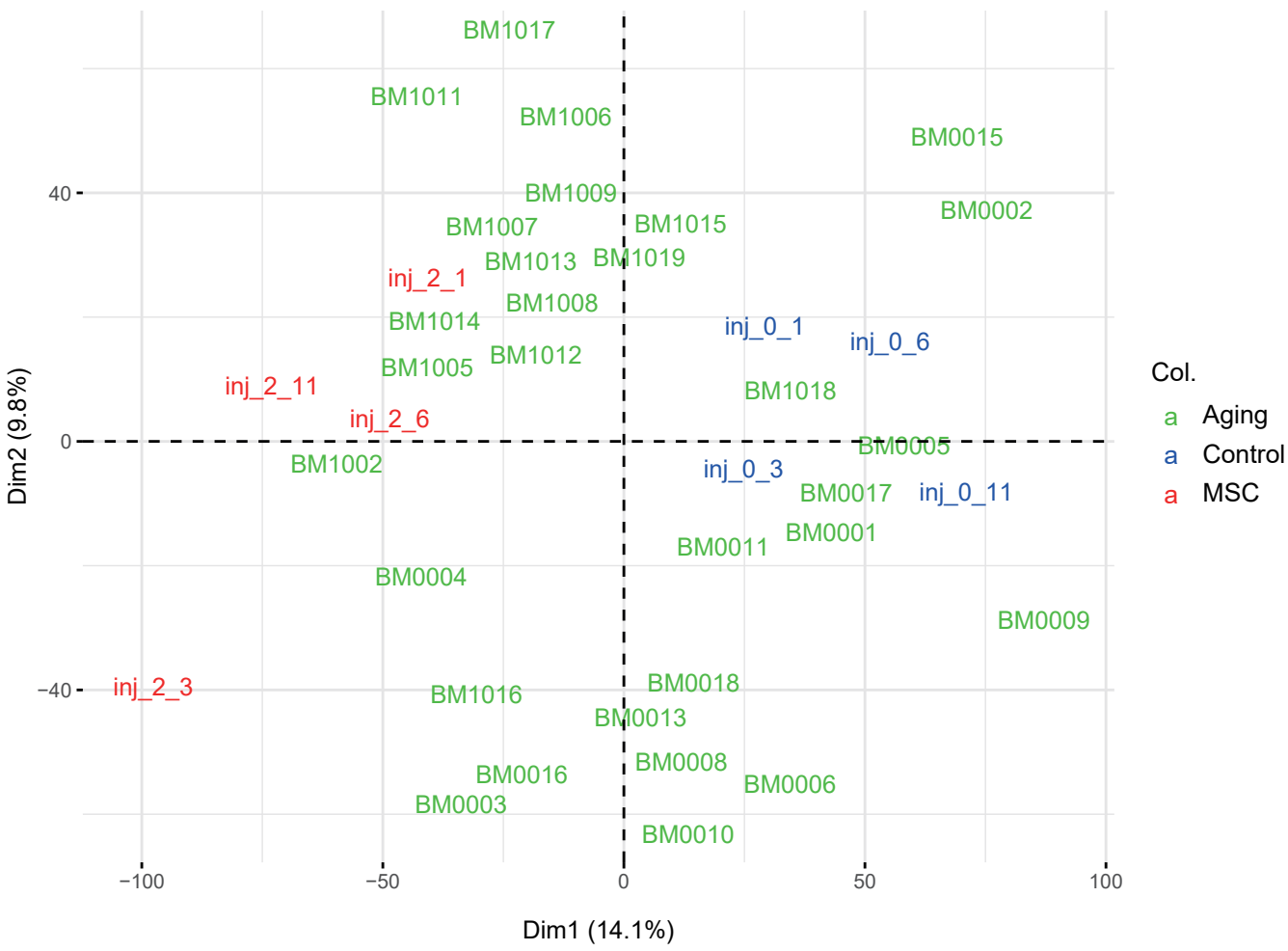

**B** Individuals – PCA

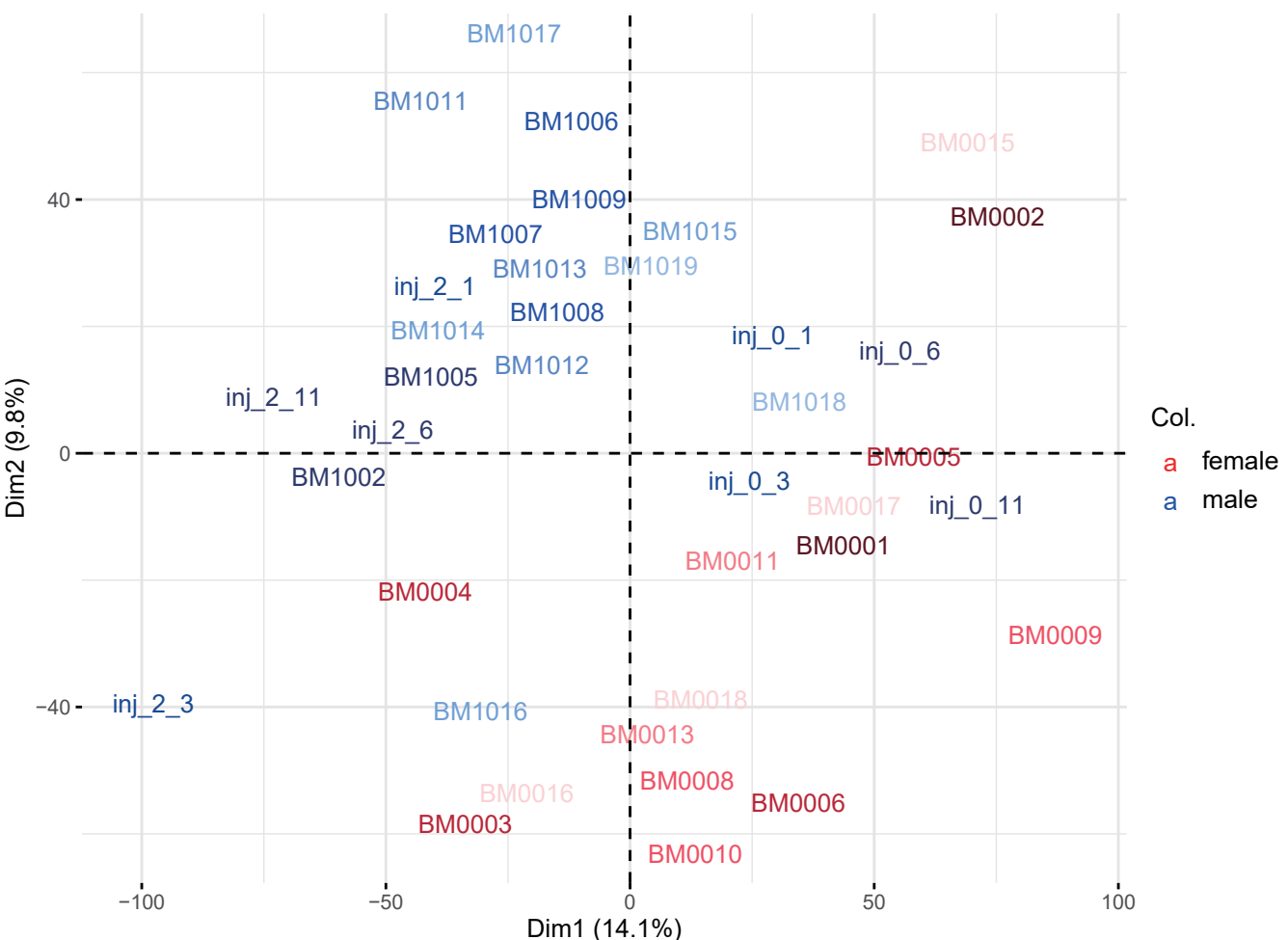

Supplement: Supplementary file 2 — Figure S2. [file ACEL-23-e14127-s004.pdf]

A

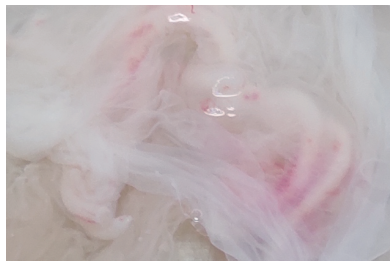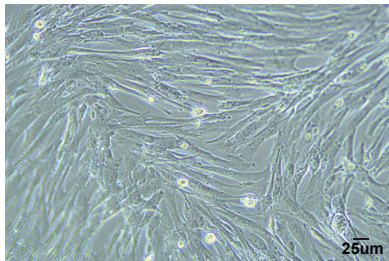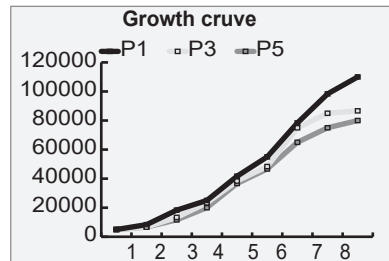

B

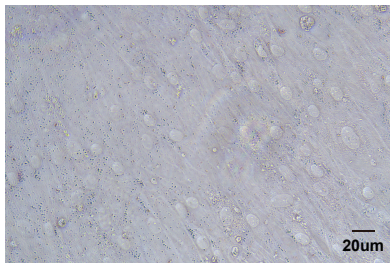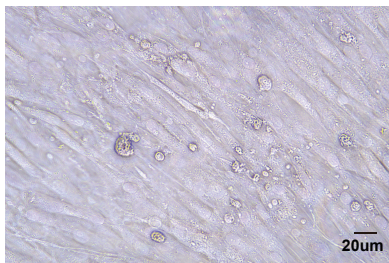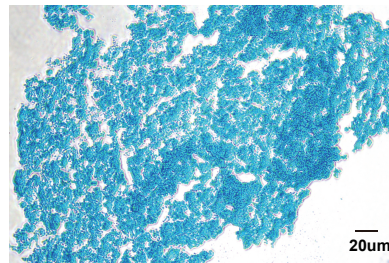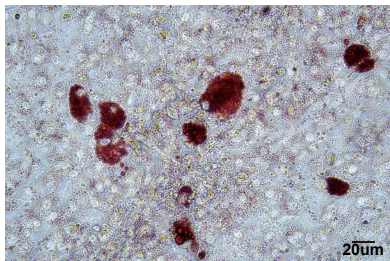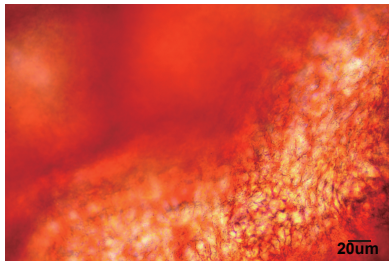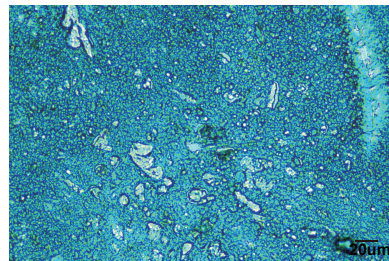

Supplement: Supplementary file 3 — Figure S3. [file ACEL-23-e14127-s014.pdf]

## Dog RNA-seq Data of Interventions

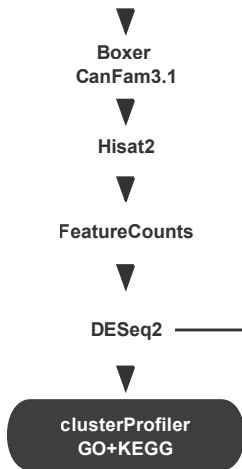

## Dog RNA-seq Data for Aging

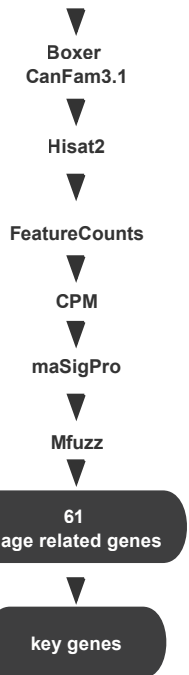

Supplement: Supplementary file 4 — Figure S4. [file ACEL-23-e14127-s012.pdf]
