# Supplementary material for: Transcriptomic and intervention evidence reveals domestic dogs as a promising model for anti‐inflammatory investigation
Source: Aging Cell. 2024 Mar 1;23(5):e14127. doi: 10.1111/acel.14127 (PMC11113267; doi:10.1111/acel.14127)
Supplement: Supplementary file 16 — Captions. [file ACEL-23-e14127-s002.docx]

#### Figure S1. Aging baseline in Malinois

1. Clusters of all genes across ages. Genes with similar expression profiles across 1, 3, 5, 7, and 9 years old were assigned to the same cluster, and the black line shows the cluster center.
2. Clusters of all genes after MSC treatment. C is the control group with dogs before injection, and MSC is the test group with dogs after injection.
3. Venn diagram of two age-associated gene identification methods.
4. Venn diagram of age-associated genes and sex DEGs. Age-associated genes identified by maSigPro were not overlapped with sex DEGs identified by DESeq2, Padj < 0.05, logFC < |1.0|.

#### Figure S2. PCA of all dog samples.

1. PCA of all dog samples. Different colors represent blood samples from dogs in different groups. “inj_0” means before the injection of MSCs, “inj_2” means after the injection of MSCs; “BM” means Belgian *Malinois* for transcriptomic aging analysis, which were not treated.
2. PCA of all dog samples colored by sex, the darker the color, the older the age.

#### Figure S3. Morphology and differentiation potential of MSCs derived from the umbilical cord.

1. Umbilical cord (UC) of dog’s perinatal tissues. Status of MSCs in primary culture to obtain full adherence. Cells appeared plastic-adherent and fibroblast-like, 200×. The growth curves at P1.
2. Results of the differentiation of MSCs. Above is the control groups, below is the experimental groups. Left to right: adipogenic (the fat droplets were stained with oil red O and showed a significant red color), osteogenic (Alizarin Red and calcium salts undergo a chromogenic reaction to produce a dark red compound) and chondrogenic (acidic mucopolysaccharides in chondrocytes were stained with Alcian Blue) differentiation, 400×.

#### Figure S4. Flowchart of the RNA-seq data analysis process

### Table legends

**Table S1. GO enrichment for 8 clusters**

Canine and human one-to-one orthologs were used as the total candidate genes (OMA Orthology Database). These genes were finally grouped into 8 clusters, and Gene Ontology (GO) terms (Top 30) for the genes in each cluster were performed separately. We noticed that the gene expression in Cluster 5 increased slowly with age and was dramatically upregulated at age 7. These genes were mainly enriched in immune-related GO terms.

**Table S2. Age-associated genes and sex DEGs**

DESeq2 and maSigPro were used to identify the age-associated genes, and DESeq2 was used to identify the sex DEGs. Venn diagram results showed that there was no overlap between age-associated genes and sex DEGs, which means that the age-associated genes that we identified were not related to sex.

**Table S3. Mean heart rate and heart rate reserve before and after treatments**

Mean heart rate (mean HRT) and heart rate reserve (HRR) data before and after MSC, NMN and rapamycin treatments. The mean heart rate (mean HRT) showed significant increases after MSC and NMN treatments, and heart rate reserve (HRR) showed significant increases after MSC treatment.

**Table S4. Concentrations of three cytokines after treatments**

Concentrations of interleukin 6 (IL-6), tumor necrosis factor alpha (TNF-α) and insulin-like growth factor-1 (IGF-1) in the plasma significantly declined after MSC, NMN and rapamycin treatments.

**Table S5. Differentially expressed genes before and after MSC treatment**

**Table S6. KEGG pathway enrichment of MSC-treated DEGs**

We performed KEGG pathway enrichment analysis of DEGs before and after MSC treatment (p.adjusted < 0.05). The upregulated DEGs were mainly enriched in the cell renewal system. The downregulated DEGs were mainly enriched in Wnt signaling pathway.

**Table S7. Potential age-associated genes**

We screened the reversal of age-associated genes by MSC-treated DEGs (p < 0.05, |log2 (fold change) | > 1). We found 3 potential aging-associated genes and plotted their expression pattern according to their z score of the normalized count.

**Table S8. Data quality of dog samples**

An average of 44.6 million high-quality (Q30 > 90%) 150 base pair reads were generated per sample.

**Table S9. The input parameter for removing batch effects**

We specified the batch variable for adjusting the batch effects, and specified ‘sex’, ‘age’, ‘breed’, and ‘group’ as biological covariates, whose signals were preserved in the adjusted data.

**Table S10. Age-associated genes identified by two different methods**

Two methods (maSigPro and ImpulseDE2) were used to identify age-associated genes (Padj < 0.05). All the genes identified by ImpulseDE2 were included in the genes identified by maSigPro.

**Table S11. Expression of canine MSC surface marker genes**

The expression of canine MSC surface marker genes was tested at passage 4 and met the criteria for cMSCs.
